# Sizing the largest ocean waves using the SWOT mission

**DOI:** 10.1073/pnas.2513381122

**Published:** 2025-09-16

**Authors:** Fabrice Ardhuin, Taina Postec, Mickael Accensi, Jean-François Piolle, Guillaume Dodet, Marcello Passaro, Marine De Carlo, Romain Husson, Gilles Guitton, Fabrice Collard

**Affiliations:** ^a^Université de Brest, CNRS, Ifremer, Institut de Recherche pour le Développement, Laboratoire d’Océanographie Physique et Spatiale (LOPS), Institut Universitaire Européen de la Mer, Plouzané F-29280, France; ^b^Deutsches Geodätisches Forschungsinstitut der Technischen Universität München, Arcisstraße 21, Munich 80333, Germany; ^c^Collecte Localisation Satellites, Plouzané 29200, France; ^d^OceanDataLab, Locmaria-Plouzane 29200, France

**Keywords:** ocean waves, 4-wave interactions, swell, SWOT, storms

## Abstract

Winds generate waves over the oceans with a wide range of properties. The largest wave heights and periods are important parameters in the design of marine structures. Extreme waves also play an outsize role in air–sea fluxes and coastal dynamics, and leave imprints on seismic and sediment records. Rare events have so far escaped measurements, with few wave heights from satellite altimeters exceeding 16 m, and no associated measurement of wave periods. Here, we use swells radiated from storms to reveal long wave periods within the storms, and their generation mechanism. Swells are resolved in the Surface Water and Ocean Topography (SWOT) satellite sea level measurements. Patterns of increasing swell wavelength and decreasing swell height away from storms are consistent with a nonlinear transfer of energy from short to long period waves. We propose an updated parametric shape for wave spectra in storms that aligns with SWOT swell measurements. It reduces energy levels by a factor of 20 at 1.2 to 1.4 times the peak period compared to commonly used spectral shapes and allows estimation of storm wave periods from swell heights. Consistent with less extreme conditions, the peak period generally increases with wave height. This was particularly verified for the largest storm peak period of 20.2 ± 0.6 s, obtained for the event with the largest significant wave height 19.7 ± 0.3 m measured by altimeters. These observations of long period swells should have a wide range of applications from coastal dynamics to seismology.

## Significance

Swells travel across ocean basins, retaining precious information about extreme storm waves that elude direct observation. Precise sea level measurements from the Surface Water and Ocean Topography satellite yield swell heights and lengths. We find that the long waves in the largest storms draw their energy from steep shorter waves, enabling storm waves to reach phenomenal heights. These long waves then radiate outward as swell. Swell measurements thus reveal properties of extreme storm waves, including their dominant period. Our analysis corrects a 20-fold overestimation in empirical expressions for the energies of the longest ocean waves, providing an awareness of ocean wave properties, with immediate applications to marine meteorology, ocean and coastal engineering, and the interpretation of ocean-generated seismic signals.

Wind-generated waves can grow to extreme sizes, affecting all activities at sea and on the coast. With little or no direct measurements of the most extreme waves, and the added complexity of climate change, there is much uncertainty on coastal hazards (1) and the safety of existing and future oceanic and coastal infrastructure (2, 3). Routine in situ wave measurements typically started in the 1980s, with few instrumented locations. Scientists and engineers have therefore used numerical models to estimate wave heights and periods for events that occur once every 30 y or more, with scarce validation for wave heights above 16 m. Various proxies of storm intensity have also been sought in sediment records, with ripple wavelengths (4) determined by both wave height and period, or the size of boulders moved during storms (5). Microseisms, another promising record of storm intensity (6, 7), indicate the presence of long-period ocean waves but lack a clear link to wave heights. In particular, it is not easy to associate ocean wave properties to long microseisms with periods up to 26 s (8) because there is no global-scale validation of numerical wave models for periods above 20 s.

**Fig. 1.**
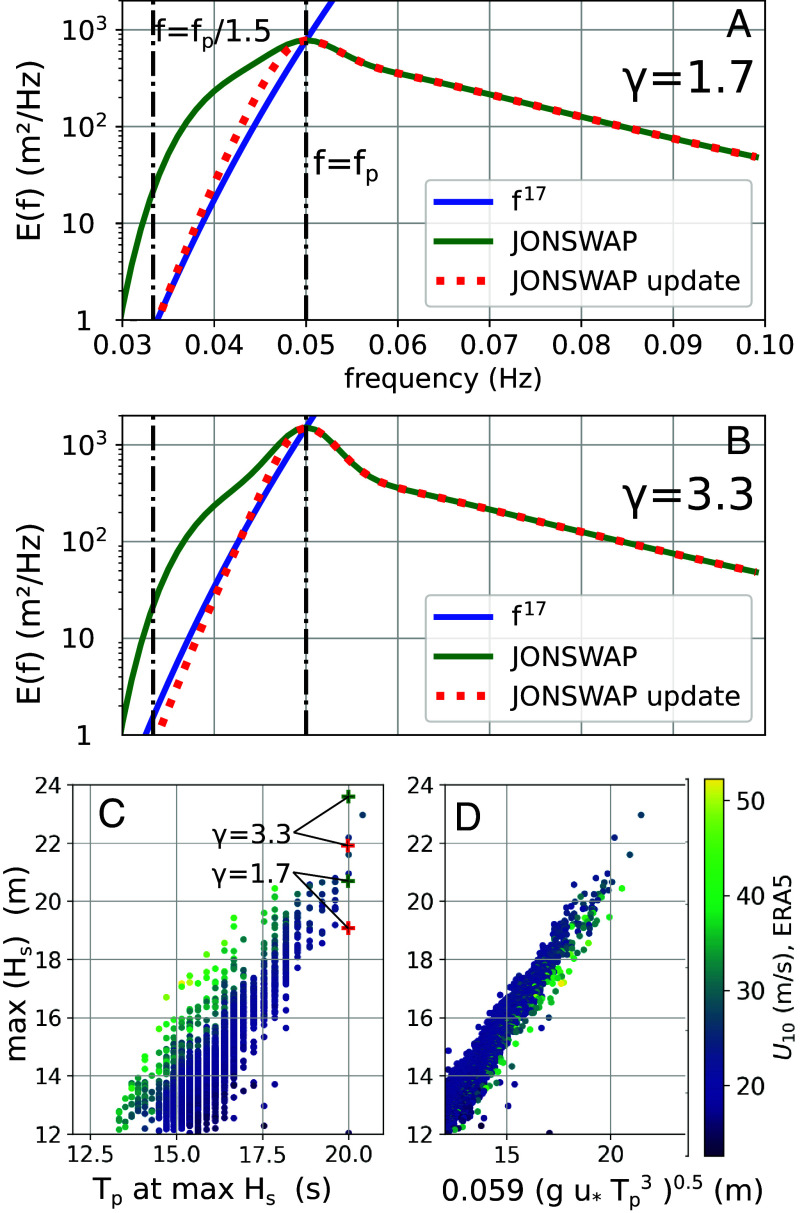
Examples of wave spectrum shape and wave growth: The Empirical “JONSWAP” form for the wave spectrum, defined by Eq. **9**, is shown for $T_{p}=20$ s and two values of the “peak enhancement” parameter γ: (*A*) γ=1.7, (*B*) γ=3.3. Our proposed updated spectral shape given by Eq. **10**. Different spectral shapes will give different relations between $H_{s}$ and $T_{p}$. (*C*) scatter diagram of modeled values (10) at the time and location of the $H_{s}$ maximum, for years 1993–2024, with colors showing the wind speed. Each dot represents one storm. “+” symbols show combinations of $H_{s}$ and $T_{p}$ from the spectral shapes in (*A* and *B*). (*D*) model data rescaled using Toba’s Law (11) with a slightly adjusted parameter: 0.059 instead of Toba’s 0.05.

The most common parameter used to quantify ocean waves is the significant wave height $H_{s}$, defined as 4 times the SD of the surface elevation, over a 20 min time series, or over a few kilometers squared when measured from space (9). The distribution of wave energy across frequencies, represented by the spectrum E(f) provides additional information needed to compute energy fluxes, or forces on structures (2). Example spectra in Fig. 1 *A* and *B* are parametric shapes adjusted to in situ datasets (12, 13), including the JOint North Sea WAve Project (JONSWAP; see *Materials and Methods*). The peak frequency $f=f_{p}$ at which E(f) is maximum, is often used to characterize the dominant waves, and corresponds to the peak period $T_{p}=1/f_{p}$. In these spectra, defined by Eq. **9**, a higher value of the shape parameter γ gives a more peaky spectrum.

Observations of ocean waves show that both $H_{s}$ and $T_{p}$ grow as the wave field develops. The two variables are correlated and this is generally well reproduced by numerical models for average conditions, for which we have measurements, but it also holds for modeled extremes (Fig. 1*C*). For a given value of $T_{p}$, $H_{s}$ may vary by ±10%, with larger wave heights corresponding to stronger wind forcing. Toba (11) proposed an empirical law that predicts $H_{s}$ from the wind speed and peak period, that is well reproduced by models (Fig. 1*D*). For example, tropical cyclones (TCs) have the highest wind speeds and for a given wave height their peak periods can be 10% shorter than those of extratropical storms (ETSs): The modeled wave spectra in TCs are more peaky than those in ETSs.

Spectral shapes are generally characterized by a well-known “tail,” for frequencies above $2f_{p}$, with a level proportional to the frequency to a power −4 to −5 (11, 14, 15). The “head” at frequencies below $f_{p}$ is little explored in spite of its contribution to $H_{s}$ (Fig. 1 *A* and *B*). Energy in this head was explained by Hasselmann (13, 16): Nonlinear 4-wave interactions transfer energy from higher frequencies, generating waves with phase speeds that can exceed the wind speed $U_{10}$. In fact, 4-wave interactions may be the only process that can generate waves with periods exceeding $2\pi U_{10}/g$, with g the acceleration of gravity, while the wind generates shorter components. It is unknown how much wave–wave interaction theory can explain wave properties in extreme conditions: High wind speeds may introduce additional effects that can modify wind–wave interactions (17) and wave breaking may also promote energy transfer to low frequencies (18). Numerical wave models are based on a dynamical balance between wind–wave generation, dissipation by breaking, and nonlinear 4-wave interactions (19). The generation and dissipation are semiempirical expressions that were adjusted to average conditions (19) and models may be less accurate in extremes.

Here, we analyze novel swell measurements (20), provided by the Surface Water and Ocean Topography (SWOT) satellite mission (21), and link these to wave spectra within extreme storms.

## Swell Dispersion, Storm Fingerprints and Wave Dynamics

### Satellites Miss the Peak of Storms.

**Fig. 2.**
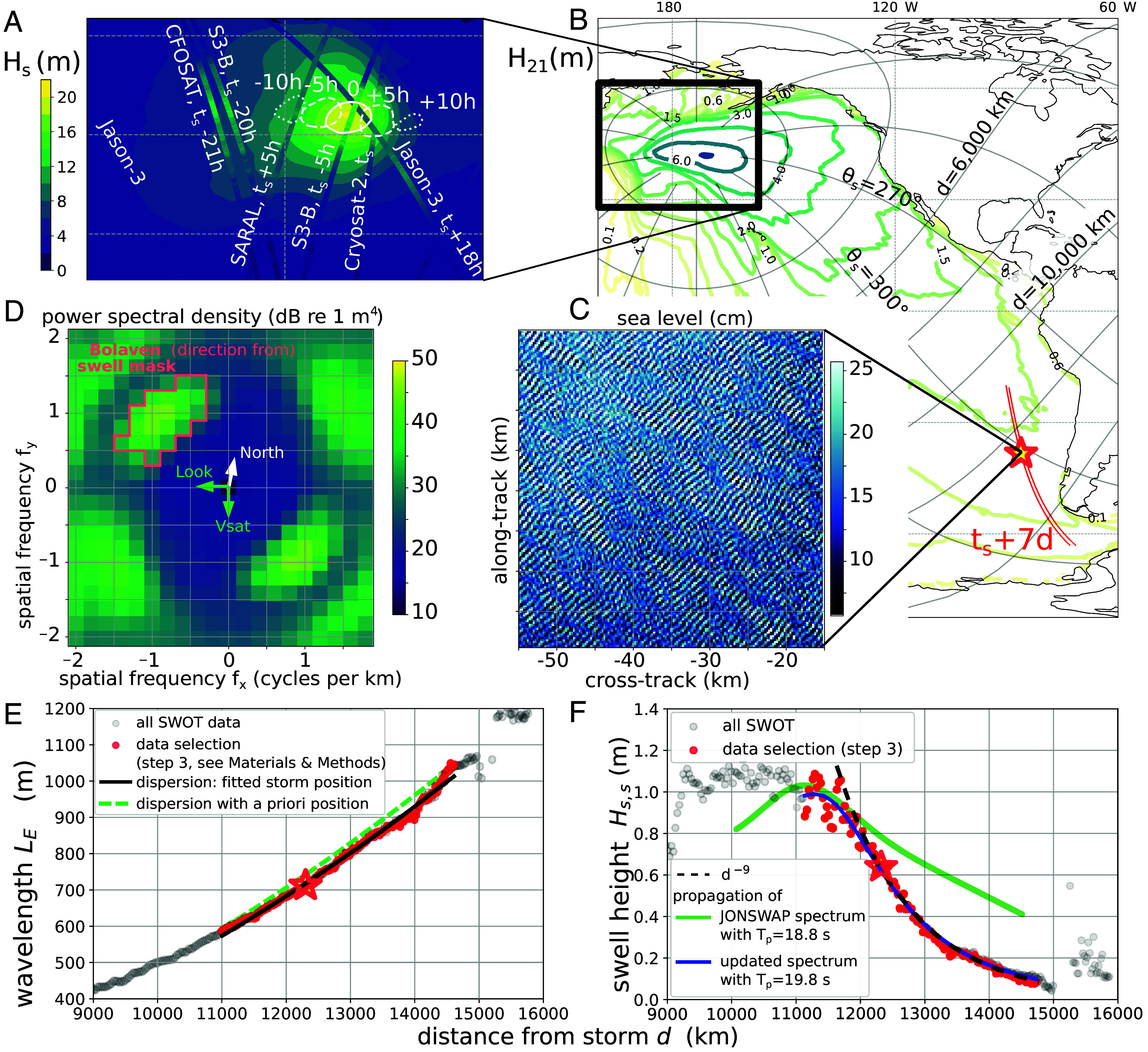
Example storm and swell field. (*A*) map of modeled wave heights at the time $t_{S}$ of the storm peak (16 October 2023, 1:00 UTC), and altimeter measurements within 20 h ($t_{S}-20$ to $t_{S}+20$h) from satellites Jason-3, Cryosat-2, SARAL, CFOSAT, Sentinel-3B. (*B*) modeled wave heights restricted to periods longer than 21 s: Contours indicate the maximum $H_{21}$ from October 15 to 27, label units are meters, and, in red, a subset of locations of SWOT measurements where Bolaven swells are observed for track 328 on 24 October. At the location of the red star, 38.1^°^S 274.6^°^E, (*C*) is an example SWOT surface elevation field, and (*D*) show the corresponding wave spectrum in which Bolaven swells are isolated in the spectral domain (red polygon). We analyze the evolution of this swell with (*E*) the mean swell wavelength $L_{E}$ and (*F*) the significant swell height $H_{\mathrm{ss}}$ as a function of distance from the storm.

In situ and satellite wave measurements are rare for wave heights over 16 m. We illustrate this with storm Bolaven, a tropical cyclone that became an intense extratropical storm in October 2023. As a reference, we use a wave model (10) that was adjusted to the highest wave heights of 2011 (22). For Bolaven, the significant wave height reached 20.3 m, the largest modeled value globally for the entire year of 2023. The modeled $H_{s}$ exceeds 18 m in a region smaller than 300 km that appears on October 15 at 12 h UTC, moves rapidly to the east, and vanishes 24 h later (Fig. 2*A*). The maximum $H_{s}$ estimated from the Sentinel-3B satellite altimeter is only 15.4±0.2 m, when sampling-induced fluctuations are properly filtered (9). At the time and location of the satellite measurement, our model gives a wave height of 17.2 m, and it is hard to know whether our model generally overestimates the large wave heights or whether there is a small time shift of the storm that also contributes to this difference. We note that on the same day, the maximum value for $H_{s}$ in the ERA5 reanalysis (23) is 15.2 m, but it probably underestimates the true wave heights, as has been often reported (24). No satellite track comes within 300 km of the maximum modeled value, and even the swath of SWOT over which $H_{s}$ can now be measured (25) is not enough to capture the peak of the storm.

Observing swells away from the storm provides a much larger region of interest, where more satellite data are available. In numerical models, we can compute a partial wave height $H_{21}$ limiting the wave energy to periods longer than 21 s, peeling off the shorter and usually higher waves to reveal long waves. Movies S1 and S2 show long wave heights starting from a short-lived and compact pulse that later disperses across half of the Pacific, and Fig. 2*B* shows that this single source dominates wave periods longer than 21 s from October 15 to 27. Separating long swells from other waves requires measurements that resolve their wavelength.

### Swells Measured by SWOT.

The SWOT satellite mission provides maps of the sea surface at 250 m resolution (Fig. 2*C*), resolving swells longer than about 500 m (26, 27), which corresponds to a deep-water linear wave period of 18 s. SWOT measurements cover two 50-km-wide swaths on either side of the satellite track. We developed a method (20) based on the Fourier transform of surface elevation maps, to obtain swell spectra (27) $E(f_{x},f_{y})$ with $f_{x}$ and $f_{y}$ the spatial frequencies in the satellite cross-track and along-track directions (Fig. 2*D*). These spectra contain few energetic peaks that correspond to swells easily associated with well-defined storm events (*Materials and Methods*).

Because SWOT can measure swell heights as low as 3 cm (20), we can follow swell “fore-runners,” those components with frequencies below the peak frequency in the storm, observed at the leading edge of the swell field (28). We use the evolution of the “energy wavelength” $L_{E}$ and swell height $H_{\mathrm{ss}}$ as a function of distance d from the storm (see *Materials and Methods*, Eqs. **1** and **2**). For propagation paths away from islands, all storms exhibit the same pattern. The wavelength increases like $d^{2}$ (Fig. 2*E*), as expected from the dispersion of linear waves (29). A more puzzling feature, previously unreported, is the sharp decrease in swell height, with a power law $d^{-n}$ with n≈9 (Fig. 2*F*). This remarkable feature is present in the hundreds of SWOT tracks that we have analyzed and is interpreted below in terms of wave properties in the storm.

### Wave Spectra in the Storm.

Swell energy is proportional to the square of the swell height and thus decays like $d^{-18}$. This energy is a spectral density multiplied by the frequency width that, in theory, varies like 1/d, and the width in direction that, neglecting the effect of currents, varies like $1/\sin(d/R_{E})$ with $R_{E}$ the Earth radius (30). Neglecting energy dissipation, theory predicts that spectral densities are conserved along the propagation path (29), as confirmed by swell measurements across the Pacific (31, 32), with weak dissipation diagnosed for periods longer than 15 s. As a result, for any azimuth, for example $\theta_{s}=300^{{}^{\circ}}$ in Fig. 2*B*, the wave spectrum in the storm $E(f,\theta_{s})$ should vary like $f^{17}$.

We note that exact numerical simulations (33, 34) of the inverse energy cascade associated with nonlinear interactions produce spectra with exponents 16 to 17 for frequencies between $0.5f_{p}$ and $0.9f_{p}$. The theory for 4-wave interactions (16) assumes a small ratio of wave height to wavelength and should apply to 20 m high waves that are 600 m long. The high exponents n inferred from SWOT swell propagations is consistent with 4-wave interactions playing a dominant role in generating energy below the spectral peak, including at lower frequencies than previously considered.

The main finding of JONSWAP (13) was that nonlinear interactions play a dominant role in wave growth, building up the peak of the spectrum. The parametric JONSWAP spectrum was thus a modification of the earlier Pierson and Moskowitz (PM) (12) shape and focused on a narrow range of frequencies around $f_{p}$. That earlier PM shape was not modified for frequencies below 0.8$f_{p}$, where we find that it is not consistent with 4-wave interactions. Other data, with high spectral resolution (35), do have large exponents, sometimes higher than 16 for $f<0.9f_{p}$, and it may be a common feature of growing wind seas. We propose to fix the PM shape, and update the “JONSWAP spectrum” for $f<f_{p}$, to include a $f^{17}$ slope and a smooth adjustment around the peak (Fig. 1 *A* and *B*; see *Materials and Methods*). This updated spectrum gives a simple asymptotic variation of swell heights that is only a function of the peak periods in the storm and the storm radius, and a much better fit to the SWOT data (Fig. 2*F*).

### Storm Peak Periods.

Fitting the variation of the swell height as a function of the distance from the storm, provides an estimate of $T_{p}=1/f_{p}$ in the storm, a parameter that we call “storm peak period” (SPP) to avoid confusion with the local swell peak period. Combining all satellite tracks for storm Bolaven (Dataset S1), we obtain SPP = 19.4 ± 0.4 s, close to the modeled value at the peak of the storm $T_{p}=19.6$ s, and consistent (11) with a wave height $H_{s}≃19$ m given by Toba’s law, much higher than the nearest altimeter measurement at 15.4±0.2 m. We note that in ERA5, the SPP is 16.1 s, consistent with lower wave heights in that model.

Our fitting method provides SPP estimates for the largest storms but it cannot apply when the dominant waves are hardly resolved (e.g. the shorter swell components in Fig. 2*D*). In those cases, we may only state that a period of 18 s, corresponding to the SWOT resolution limit, is an upper bound for the SPP.

## Consistency of Extreme Wave Parameters from SWOT

**Fig. 3.**
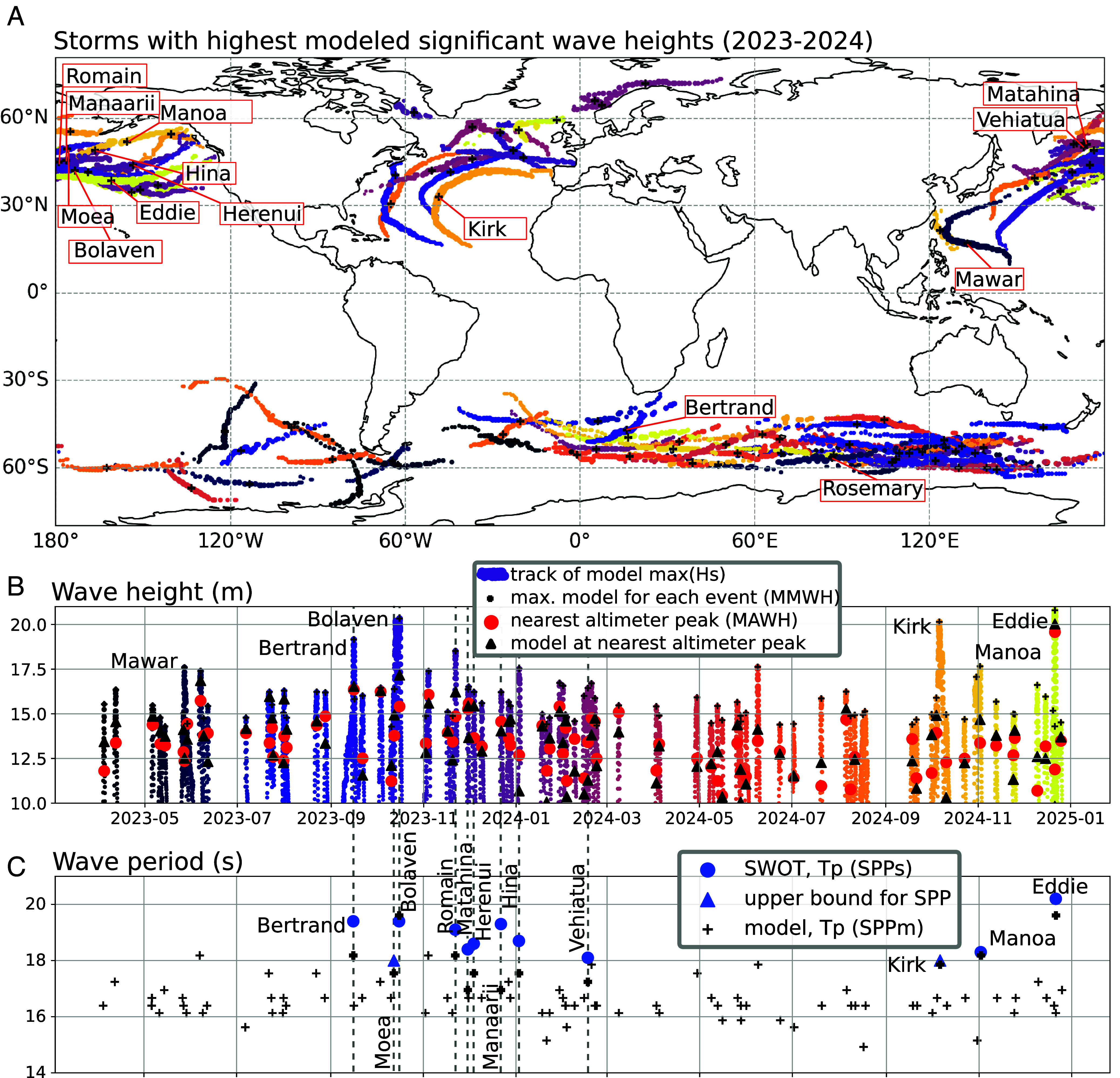
Storm tracks, maximum Hs, and storm peak periods over the SWOT era. (*A*) map of the 100 most severe storms from April 2023 to December 2024, with the tracks of the most severe storms plotted on top of the weaker ones. Storm tracks are provided by a numerical model (10) and colored by time. (*B*) time series of modeled $H_{s}$ along the tracks [same colors as in (*A*)], and corresponding highest altimeter $H_{s}$ (*C*) storm peak periods (SPP, in seconds) at the location of the storm $H_{s}$ maximum.

The indirect method proposed above is hard to validate because of the lack of measurement in extreme conditions. However, we can check the consistency of our storm peak period estimates using altimeter data and expected relations between wave heights and periods. Instead of using wind or atmospheric pressure to define storms (36–38), we have chosen to identify storms from the tracks of wave height maxima (39). This also provides the location from which the swells radiate (Fig. 2 *A* and *B*). To simplify the discussion, we gave names to storms with no name (Fig. 3, Table 1).

**Table 1.** Properties of the top 5 storms ranked by modeled maximum significant wave height (MMWH), for the years 1991–2024, and some of the largest storms for 2023–2024

Rank	Name	Date	Lon.(^°^)	Lat.(^°^)	*U_10_*	Dir.(^°^)	MMWH (m)	Model *T_p_* (s)	SWOT SPP (s)	MAWH (m)	MSLA (m)
1	Ronadh*	20140105	031W	47N	31.8	258	23.0	20.4		18.7 ± 0.2	18.4
2	Paul*	20130115	166E	39N	29.9	346	22.2	20.0		15.8 ± 0.2	16.8
3	Yoshiaki*	19981026	177W	42N	32.1	249	21.6	20.0		12.6 ± 0.2	15.4
4	Luigi*	20150427	138E	56S	28.9	239	21.0	20.0		18.1 ± 0.3	19.6
5	Eddie*	20241221	161E	39N	28.9	273	20.8	19.6	20.2 ± 0.6	19.7 ± 0.3	20.2
12	Bolaven	20231016	174W	42N	30.0	264	20.3	19.6	19.4 ± 0.4	15.4 ± 0.2	17.2
17	Kirk	20241006	49W	33N	39.4	175	20.1	17.9	<18		
56	Bertrand*	20230915	16E	47S	30.6	244	19.2	18.2	19.4 ± 0.3	16.4 ± 0.2	16.5
92	Romain*	20231122	179W	45N	29.2	267	18.5	18.2	19.1 ± 0.3	15.0 ± 0.2	16.1
192	Manoa*	20241102	156W	52N	27.4	252	17.7	18.2	18.3 ± 0.1	13.4 ± 0.2	14.7
206	Mawar	20230527	133E	17N	43.1	77	17.6	16.4		12.7 ± 0.1	12.5
234	Rosemary	20230606	87E	57S	26.2	254	17.4	18.2		15.7 ± 0.2	16.8
489	Moea*	20231012	176W	50N	26.4	255	16.4	17.5	<18	13.7 ± 0.2	14.9
569	Manaarii*	20231222	177W	46N	27.3	263	16.3	17.5	19.3±0.2	14.6 ± 0.1	14.0

Storm names with an asterisk are preliminary and were given by the authors and the rank corresponds to the order in the 1991–2024 ADC storm catalog (39). Date, longitude, latitude and wave direction “dir” (mean wave direction from where the waves propagate) are given for the MMWH. MAWH stands for maximum altimeter $H_{s}$, MSLA is the model $H_{s}$ at the time and location of MAWH, which differ from the time and location of MMWH.

The most severe events for 2023 and 2024 follow the usual storm tracks in the North Pacific and North Atlantic (Fig. 3*A*). Kirk and Bolaven are tropical storms that reintensified as extratropical storms. A larger number of events occurred in the southern ocean but, according to the model, they generally have lower maximum wave heights. To verify these model results, we searched for maximum altimeter wave heights (MAWH) within 1,000 km and 24 h of the model maximum wave height (MMWH). This dataset (40) includes eight satellites in 2023: SARAL, Jason-3, Sentinel-3A, Sentinel-3B, Sentinel-6, Cryosat-2, CFOSAT, and SWOT. In 2024 we only use data from CFOSAT and SWOT. Fig. 3*B* confirms that altimeters generally miss the storm peak and give MAWH that are on average 2.2 m lower than the MMWH, two quantities that are poorly correlated (r = 0.59). The MAWH is a lower bound on the storm maximum wave height but a poor indicator of storm maximum wave height. The model at the same location as the altimeter (MSLA) is highly correlated with the satellite value at the same location (r = 0.98, bias = 0.3 m, rms difference = 0.95 m). This is an important statistical validation of our wave model (10) for extremes.

Starting in April 2023, SWOT provides a complementary measure of storm wave properties, with observations of long swells from all of the most severe storms (41). We have analyzed 10 North Pacific storms that are easy to interpret because swell propagation is little impacted by islands.

We named the most intense event “Eddie:” This extratropical storm produced large waves in Hawaii for the big wave surfing competition “the Eddie” and caused casualties and extensive damage on the American coasts from Canada to Peru. At a distance of 5,000 km from the storm center, SWOT measured swells higher than 0.2 m with a mean wavelength exceeding 1,200 m, which corresponds to a really long period of 28 s. Further away, at 5,600 km from the storm center, swells with a mean wavelength of 1,360 m (i.e. a period of 30 s) and a height of 6 cm are near the SWOT detection limit. This sharp decay in wave height is again close to $d^{-9}$ (*SI Appendix*, Fig. S1).

Eddie is a very interesting case for assessing the consistency of our method because it had the longest SPP with 20.2±0.6 s and altimeter measurements close to the storm center, at 8:55 UTC on December 21, only 6 h before the storm maximum wave height was obtained in our model. For that storm we are sure that the significant wave height exceeded 19.7±0.3 m as measured by the Poseidon-3C altimeter on board the SWOT satellite. This measured value is higher than the maximum ERA5 estimate of 16.8 m with a SPP of 16.6 s, and slightly lower than our model value 20.2 m at the time and location of the maximum altimeter value, while our maximum model value is 20.8 m (10). Based on our model overestimation, we can expect that the true maximum $H_{s}$ is between 19.7 and 20.2 m. Including our update to the JONSWAP spectrum with usual values of γ between 1.7 and 3.3 (Fig. 1), these values of $H_{s}$ give a peak period that may vary between 19 and 20.5 s, consistent with our SPP estimate. Finally we note that this is the largest ever reported wave height measurement from any satellite altimeter since 1991.

In general, for a given wave height, shorter storm peak periods correspond to higher wind speeds (11), as in hurricane Kirk, and this explains that our model with a small bias for MMWH also has a small bias for SPP. Ocean currents may also have some influence, and the larger SPP value for Bertrand, compared to the model output, could be related to a model underestimation of wave–current interactions in the Agulhas region (42). Further analysis of other parameters, such as storm translation speed and wind azimuth relative to dominant wave direction, may be needed to understand SPP differences over 1 s, including the higher value for storm Manaarii (Fig. 3, Table 1).

### Recent Storms in Perspective.

So far the largest published open ocean measurement (9) of the significant wave height was for the 2011 storm Quirin (ranked 15th in our storm catalog) with $\text{max}(H_{s})=18.5\pm 0.3$ m, a value that models typically exceed twice a year (39). The lack of higher values in the data record is caused by high waves occurring in small ocean areas that are missed by satellite tracks and buoys, as was the case for Bolaven. Swell fields are not so easily missed. When sorted by modeled maximum wave height, SWOT has observed swells from 10 of the top 250 storms of the past 34 y (Table 1), and we could quantify the storm peak period for 6 of these. Within the model output, the number of these extreme storms suggests that 2023 and 2024 are statistically consistent with the wider 1991–2024 time frame. Given the large interannual variability in extreme wave heights, a meaningful analysis of trends for extremes will require longer time series.

Complementary swell measurements by CFOSAT may be used to extend our record of SPPs to 2019 (*SI Appendix*, Fig. S2). Before 2019, our knowledge of past storms can benefit from SWOT in other ways. Swell properties are measured near seismic stations that have operated for over a century (6), providing a possible calibration of the transfer function from microseism amplitudes to wave heights. This could help reconcile diverging trends in microseism and ocean wave energy (7, 43).

## Conclusions

Satellite altimetry has provided a sparse sampling of the ocean wave field since 1991, poorly capturing extreme events and measuring only wave heights. Using the unique capability of SWOT to measure long swells, we have analyzed swells from the largest storms from April 2023 to December 2024. These measurements provide an awareness of ocean wave properties. For example, exceptional swells can exceed wavelengths of 1,200 m, which corresponds to a really long period of 28 s. Although such long swells were anticipated by Munk (44) who predicted that measurable wave components may be found at twice the dominant period, the properties of waves much longer than the peak period had, so far, received little attention. In the case of SWOT data, which are limited to wavelengths longer than 500 m and a swell height larger than 3 cm, we generally find waves up to 1.5 times the storm peak period, defined as the peak period at the time and location where the significant wave height is maximum.

SWOT data and simple linear propagation suggest that standard parametric spectral shapes (13), used in all engineering applications (2), overestimate low frequency energy and should be revised. We propose an updated spectrum that is consistent with the idea that nonlinear energy transfer (16) from dominant waves is the main source of longer period waves. This link between dominant waves in the storm and longer swells allows us to estimate storm peak periods in extreme extratropical storms, with more difficulties for the shorter periods of tropical cyclones, due to the limited 250 m resolution of SWOT low rate data. For example, the north Pacific storm “Eddie” had its largest waves on December 21, 2024, with a maximum significant wave height of at least 19.7 ± 0.3 m and a corresponding peak period of 20.2 ± 0.6 s.

Inversely, the possible swell heights and periods can be estimated from a few storm properties, which should be useful in a wide range of engineering and geophysical applications for which wave periods are essential parameters. These include the design of structures and adaptation to climate change, in particular in coastal areas, but also the interpretation of sedimentary records (4) and the analysis of yet unexplained seismic signals, at periods 18 to 26 s (8), possibly associated with ocean waves of the same periods.

## Materials and Methods

### Swell Partitions in SWOT Spectra and Associated Heights *H_ss_* and Wavelengths *L_E_*.

Surface elevation spectra were computed from 40 by 40 km “boxes” every 40 km centered along both red lines in Fig. 2*B*. For each box, the spectrum is the average of the modulus square of the Fourier transform over 5 by 5 km tiles (27), giving 128 degrees of freedom.

A “swell partition” associated to any given storm is isolated by masking the spectrum, keeping only those spectral components that could reasonably come from the storm: Namely, we compute the spatial frequencies $\left(f_{\mathrm{xc}},f_{\mathrm{yc}}\right)$ that correspond to waves from the first guess source time and position, and the swell mask is defined by: 1. keeping spectra components that have spatial frequencies within 30% of $f_{c}=\left|f_{\mathrm{xc}},f_{\mathrm{yc}}\right|$ or that correspond to source locations within 1,000 km of the storm peak. 2. keeping spectral components that have a propagation direction within 27^°^ from the expected arrival direction.

We have visually inspected the resulting spectra and partitions to make sure that the mask boundary (e.g., red line in Fig. 2*D*) does not cut through a spectral peak.

From the masked spectrum, the swell height is defined as

[1] $$\begin{array}{c} H_{\mathrm{ss}}=4\sqrt{2E_{\text{mask}}}, \end{array}$$

where the factor two corrects for the two-sided nature of the spectrum, and $E_{\text{mask}}$ is the sum of the masked energy. The energy wavelength $L_{E}$ isd defined as gives

[2] $$\begin{array}{c} L_{E}={\left[\int_{\text{mask}}\frac{E(f_{x},f_{y})}{{\left(f_{x}^{2}+f_{y}^{2}\right)}^{1/4}}\text{d}f_{x}\text{d}f_{y}/E_{\text{mask}}\right]}^{2}. \end{array}$$

### Propagation of Analytical Spectral Shapes on the Sphere.

We use $R_{E}$ for the Earth radius, and α is the spherical distance between the storm center S and the observation point O, so that a distance along a great circle is $d=\alpha R_{E}$. We consider observations at time $t_{O}$ at a location of colatitude and longitude $\left(\lambda_{O},\varphi_{O}\right)$. The total wave energy at point O is the sum over the wave spectrum and can be rewritten as an integral of spectral densities at a chosen time $t_{S}$ and at points P in the source region, with coordinates $\left(\lambda_{P},\varphi_{P}\right)$. Using the conservation properties of spectral densities (29, 30) gives

[3] $$\begin{array}{c} E_{O}=\int_{\theta_{1}^{\prime }}^{\theta_{2}^{\prime }}\int_{f_{1}}^{f_{2}}E\left(\lambda_{P},\varphi_{P},t_{S},f,\theta^{\prime }\right)dfd\theta^{\prime } \end{array}$$

with a one to one relationship between the spherical distance $\alpha^{\prime }$ from any source location P and the observation point O, and the wave frequency f,

[4] $$\begin{array}{c} f=g\left(t_{O}-t_{S}\right)/\left(4\pi \alpha^{\prime }R_{E}\right). \end{array}$$

We can now use different simplifying assumptions. The most simple case is that of a circular storm of radius r in which the wave spectrum is uniform and isotropic at time $t_{S}$

[5] $$\begin{array}{c} E\left(\lambda_{P},\varphi_{P},t_{S},f,\theta \right)=E_{S,\text{iso}}\left(f\right)/\left(2\pi \right). \end{array}$$

This gives the asymptotic form (*SI Appendix*, section 2)

[6] $$\begin{array}{c} E_{O}\left(\alpha ,\text{SPP},r\right)=fE_{S,\text{iso}}\left(f\right)\frac{{\left(r/R_{E}\right)}^{2}}{2\alpha \;\sin\;\alpha } \end{array}$$

We use Eq. **6** to fit swell heights $H_{\mathrm{ss}}=4\sqrt{E_{O}}$ to the measurements, and obtain an estimate of SPP and storm radius r. Note that we have not interpreted the radius r because a more complete model should take into account swell dissipation (32).

### A Low-Frequency Update to Usual Spectral Shapes.

The dimensional analysis by Phillips (45) suggested that the high frequency part of the spectrum should follow

[7] $$\begin{array}{c} E_{P}\left(f\right)=\alpha_{P}g^{2}f_{p}^{-5} \end{array}$$

with $\alpha_{P}=0.0081/{\left(2\pi \right)}^{4}$ the Phillips’ constant. Measurements at sea compiled by Lionel Moskowitz have led to an empirical spectral for fully developed seas that provides a roll-off for low frequencies in the form of equation 12 in ref. 12,

[8] $$\begin{array}{c} E_{\text{PM}}\left(f\right)=E_{P}\left(f\right)\;\text{exp}\left(-1.25{\left(f/f_{p}\right)}^{-4}\right). \end{array}$$

Eq. **8** is known as the “Pierson-Moskowitz” spectrum. It was later modified (13) to also represent younger waves,

[9] $$\begin{array}{c} E_{J}\left(f\right)=E_{\text{PM}}\left(f\right)\gamma^{\exp\left(-\frac{{\left(f-f_{p}\right)}^{2}}{2\sigma^{2}f_{p}^{2}}\right)}, \end{array}$$

where the “peak enhancement factor” was found to be γ=3.3 for fetch-limited conditions. The width σ of this peak region was set to σ=0.07 for $f<f_{p}$ and σ=0.09 for $f>f_{p}$, suggesting that the peak should be steeper on the low frequency side of the spectrum. Following a common practice, we have used σ=0.07 on both sides of the peak.

We propose to keep the part of the PM spectrum for frequencies above peak (for $f>f_{p}$) and replace $E_{\text{PM}}$ with the following update for $f<f_{p}$,

[10] $$\begin{array}{c} E_{\text{PM},u}\left(f\right)={\text{e}}^{-1.25}E_{P}\left(f\right){\left(\frac{f}{f_{p}}\right)}^{5+n\;\tanh\left(\frac{f_{p}-f}{0.2f_{p}}\right)} \end{array}$$

In practice, we use n=17, and the classical JONSWAP spectrum is often used with γ=1.7 for the mature wind sea conditions considered here (14, 46). Since our updated spectral shape reduces the total energy (Fig. 1*A*), we have partially compensated this effect by using a constant γ=2. Alternatively γ might be fitted on the SWOT data, but it is sensitive to a range of frequencies where few data are available. Our estimates of the storm peak period are not very sensitive to the choice of γ, with SPP = 19.2 s for γ=1.3 and SPP = 19.3 s for γ=2 in the case of storm Bolaven. We also note that for $f>f_{p}$, which is not relevant here except for a minor effect on the wave height, more accurate shapes have been proposed (14, 15, 35, 46).

### Estimation of the Storm Peak Period (SPP): A 4-Step Algorithm.

Step 1: based on the storm catalog (39) an a priori distance to storm d and time from storm $t_{O}-t_{S}$ is defined for each swell observation. The analytical dispersion Eq. **4** with f replaced by $\sqrt{g/2\pi L_{E}}$ is fitted to the measured $L_{E}$, which provides a refined storm time and along-track position that is used in the next steps, with an updated value d of the distance to storm.

Step 2: We select the longest set of consecutive measurements of $H_{\mathrm{ss}}$ along the track, with $L_{E}$ in the range [500 m, 1,050 m]. This range is extended toward higher and lower wavelengths provided that $L_{E}$ is within 3% of the expected value (based on the analytical fit) for low values of $L_{E}$. For the large wavelength we use a more relaxed threshold of 7.5% of the expected value, and only keep the data for which $H_{\mathrm{ss}}$ decreases as a function of distance and is larger than 10 cm. A first fit of SPP and r is performed using this set of swell height and Eq. **6**.

Step 3: Fitting a wider range of wavelengths generally produces slightly lower peak periods (*SI Appendix*, section 3). Therefore, if the wavelength corresponding to SPP ($L_{\text{SPP}}=g{\text{SPP}}^{2}/2\pi$) is larger than the lowest $L_{E}$ used in the $H_{\mathrm{ss}}$ fit of step 2, then we restrict the data range to only have $L_{E}$ higher than $L_{\text{SPP}}$ and perform a second fit of $H_{\mathrm{ss}}$ using Eq. **6**. This provided the final estimate of SPP, listed in Dataset S1 and used in Fig. 3. The automatic processing of all satellite tracks is performed by the notebook L3_fit_all_tracks_LandH.ipynb.

Step 4: The quality of the fit is summarized in a Mean Absolute Percentage Error (MAPE) between observed and fitted values. If there is no peak in the fitted swell heights after Step 2 then the SPP estimate is “not successful.”

All satellite tracks data listed in *SI Appendix* were processed with the same settings except for the following adjustment: For storms Kirk and Solveig, the thresholds that define the partition in the spectrum were adjusted to avoid combining swells from 2 different storms: We used a relative tolerance of 20% (instead of 30%) on the values of $f_{c}$ included in the swell partition.

## Supplementary Material

Appendix 01 (PDF)

Dataset S01 (XLSX)

Movie S1. Visualization of modeled wave heights *H*_18_ (i.e. corresponding to the range of wavelengths L > 500 m typically resolved in SWOT Low Resolution data) and SWOT satellite tracks. The threshold at 5 cm is expected to be close to the threshold for swell detection by SWOT in most conditions. The model used here is described in ref. 15. Storm tracks for the top 400 storms, and Hs max values are overlaid over the map of *H*_18_. The black crosses along the tracks indicate the position of the maximum along the track (one example is Fig. 2A). The animation illustrates how the generation of long period waves, in the model, occurs very close in time to the maximum *H_s_*.

Movie S2. Visualization of modeled wave heights *H*_25_ (i.e. corresponding to the range of wavelengths *L* > 975 m typically resolved in SWOT Low Resolution data) and SWOT satellite tracks. The threshold at 5 cm is expected to be close to the threshold for swell detection by SWOT in most conditions. The model used here is described in ref. 15. Storm tracks for the top 400 storms, and Hs max values are overlaid over the map of *H*_25_. The black crosses along the tracks indicate the position of the maximum along the track (one example is Fig. 2A). The animation illustrates how the generation of long period waves, in the model, occurs very close in time to the maximum *H_s_*.

## Data Availability

The data generated in this study are included in: F.A., T.P., Storm tracks, wave heights and peak periods for “phenomenal” sea states, combining model, altimeter wave heights and swells measured by the SWOT satellite mission. Version 1. SEANOE (2025), https://doi.org/10.17882/105447. This is based on SWOT L2 ocean data Version C was downloaded from AVISO in January 2025. Codes generated for data processing and plotting are included.
