# Supplementary material for: Sizing the largest ocean waves using the SWOT mission
Source: Proc Natl Acad Sci U S A. 2025 Sep 16;122(38):e2513381122. doi: 10.1073/pnas.2513381122 (PMC12478040; doi:10.1073/pnas.2513381122)
Supplement: Supplementary file 1 — Appendix 01 (PDF) [file pnas.2513381122.sapp.pdf]

Supporting Information for

Sizing the largest ocean waves using the SWOT mission

Fabrice Ardhuin, Taina Postec, Mickael Accensi, Jean-François Piolle, Taina Postec, Guillaume Dodet, Marcello Passaro, Marine De Carlo, Romain Husson, Gilles Guitton, Fabrice Collard

Corresponding Author name.
E-mail: ardhuin@ifremer.fr

This PDF file includes:

Supporting text
Figs. S1 to S4
Legends for Movies S1 to S2
Legend for Dataset S1
SI References

Other supporting materials for this manuscript include the following:

Movies S1 to S2
Dataset S1

Supporting Information Text

1. CFOSAT data analysis. In addition to SWOT data, we have tested our analysis method with the 10° incidence off-nadir beam of the SWIM instrument on the China France Ocean Satellite (CFOSAT). CFOSAT was launched in 2018 and SWIM resolves waves down to 50 m wavelength, using a real aperture radar with rotating beams (1). We processed Level 2S data produced by Ifremer(2), which gives swell height, wavelength, and direction, including an empirical modulation transfer function (MTF) that provides a more accurate estimate of the wave energy compared to theoretical theoretical MTFs used in other data products and that only account for tilt effects.

For wavelengths above 800 m, SWIM is able to detect swells with heights above 0.2 m (Fig. S2). The SWIM swell heights have a larger scatter than the SWOT data, probably due to fluctuations in the modulation of short waves by long waves that define the SWIM radar measurement from which swell heights are estimated(2). In this preliminary test for Bolaven storm, we obtained SPP values that ranged from 18.8 to 19.8 s depending on the data selection criteria. Using an uncertainty model for the SWIM swell heights, one should obtain useful storm peak periods estimates, allowing an extension of our catalog back to 2019.

2. Derivation of asympototic swell height for a circular storm. We recall that the frequency and distance α' (see Fig. S3) are related by propagation at the group speed,

$$f = g(t_O - t_S)/(4\pi\alpha' R_E). \tag{S1}$$

We define $\Delta\theta' = \theta'_2 - \theta'_1$ as the range of azimuth angles over which the storm is seen from O, this is a function of the actual distance α', or equivalently, using eq. [S1] the wave frequency. Without loss of generality we consider coordinates such that the storm center S is at the south pole (Fig. S3). The spherical law of cosines in the triangle OPS, with P on the edge of the storm yields

$$\cos(\Delta\theta'/2) = (\cos(r/R_E) - \cos\alpha\cos\alpha')/\sin\alpha\sin\alpha'. \tag{S2}$$

The observed energy is now a simple sum over frequencies f that are related by eq. [S1] to the distances α'

$$E_O = \int_{f_1}^{f_2} \frac{E_{S,\mathrm{iso}}(f)}{2\pi}\Delta\theta' df = \int_{\alpha_1}^{\alpha_2} \frac{E_{S,\mathrm{iso}}(f)}{2\pi}\Delta\theta' \frac{df}{d\alpha'} d\alpha' \tag{S3}$$

This integral can be evaluated numerically using any analytic expression for the source spectrum, which can be the JONSWAP spectrum (3) or the update proposed here.

In the limit $|\alpha' - \alpha| \ll \alpha$ and $r \ll R_E$, which is appropriate far from the storm, we find, when averaged over α',

$$\Delta\theta' \simeq \pi(r/R_E)/(2\sin\alpha). \tag{S4}$$

Also, eq. [S1] gives

$$df/d\alpha' \simeq f/\alpha. \tag{S5}$$

These two asymptotic expressions are used to obtain eq. [4] in the Materials and Methods section.

3. Propagation combining different analytical spectra for different source regions. We used numerical simulations of eq. [S3] to evaluate the impact of different effects on the fitted peak periods: dissipation, non-uniformity of the source region, error in the source position. Fig. S4 illustrates the typical effect of a smaller area with larger wave heights and periods, surrounded by a wider region with lower wave heights and periods. The resulting distribution of swell heights has two maxima: the longer waves travel faster and thus dominate the swell field at larger distances. Fitting only the peak that is further from the storm gives a more accurate estimate of the longest periods (19.9 s instead of the input 20 s in Fig. S4.c) and fitting a wider range of distances / wavelengths tends to reduce the fitted period (19.1 s in Fig. S4.a).

These simulations have led us to add step 3 in our algorithm to estimate SPP. We have also verified with the numerical integration of eq. [S3] that a realistic dissipation has no significant impact on the estimate of SPP.

4. Complementarity of satellite an buoy data. It may be surprising that buoy data has not been used to measure the sharp increase of swell height in time series that corresponds to the sharp decay of swell height along the satellite track. In SI Appendix Fig. S4, the drop of H_{ss} from 3 to 1 m occurs over 500 km, which corresponds to a propagation time of 9 hours. Inspection of wave spectra from buoys off the California coast gives a rise in H_{18} from under 1 m to over 3 m in a similar duration of 6 hours (from 16:00 to 22:00 on December 23 2024 for the National Data Buoy Center number 46011 off Point Arguello), at 3600 km from the a priori center of the Eddie storm. However, buoy spectra estimated over 1 hour have fewer degrees of freedom than SWOT-derived spectra over 40 km, giving larger uncertainty on wave height estimates (4, 5). As a result, using time series for the same time of analysis would require many more buoys with higher resolution spectra than what is currently available.

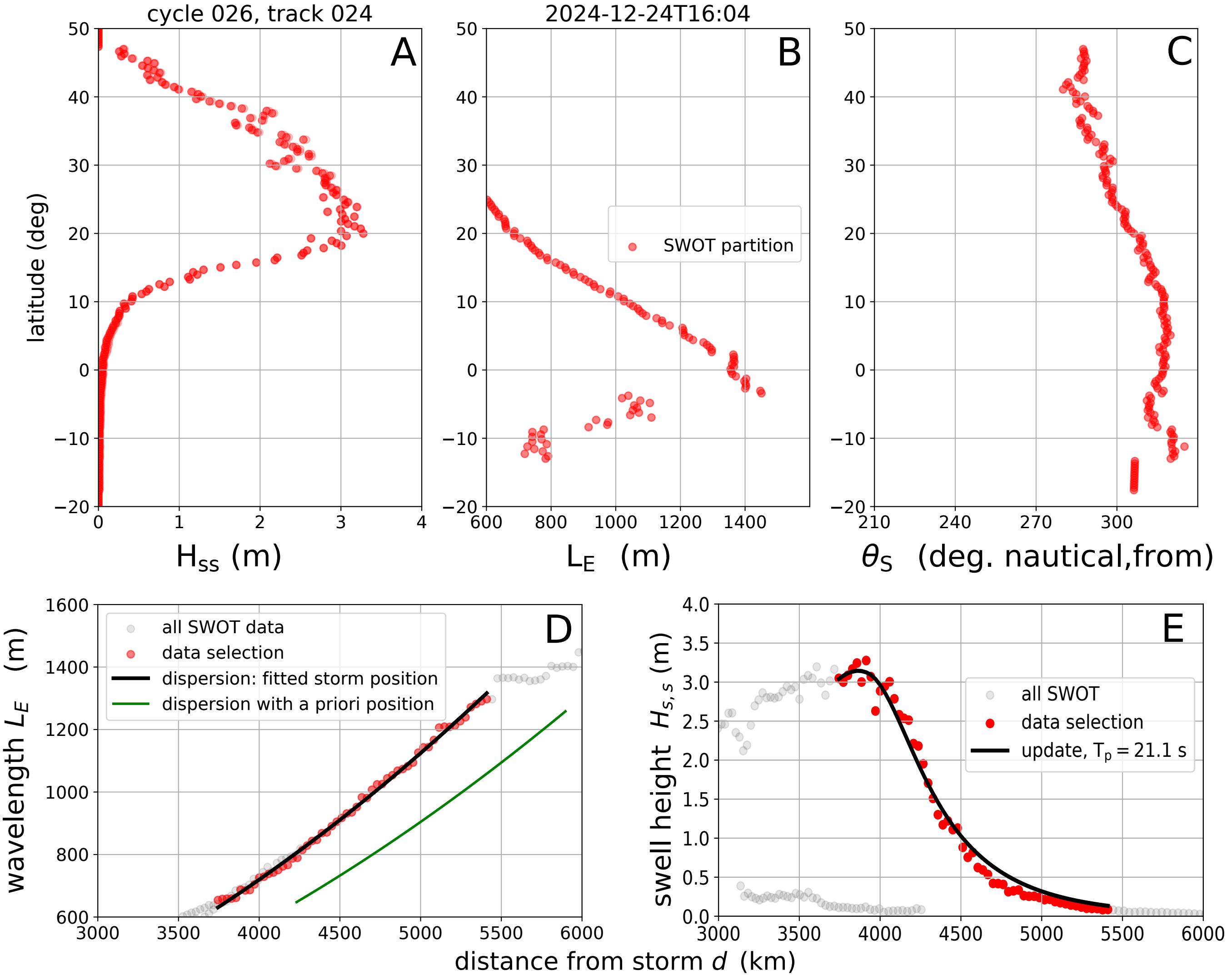

Fig. S1. Swells from storm Eddie as captured by SWOT on cycle 026 track 024 on 24 December 2024. A-C show partition heights H_{ss}, wavelengths L_E and mean direction as a function of latitude. D and E are similar to Fig 2.E and 2.F. Here the data selection was forced to use L_E in the range [650 ,1300]. Note that the discontinuous behaviour of L_E for $L_E > 1200$ m is caused by our use of a coarse wave spectrum

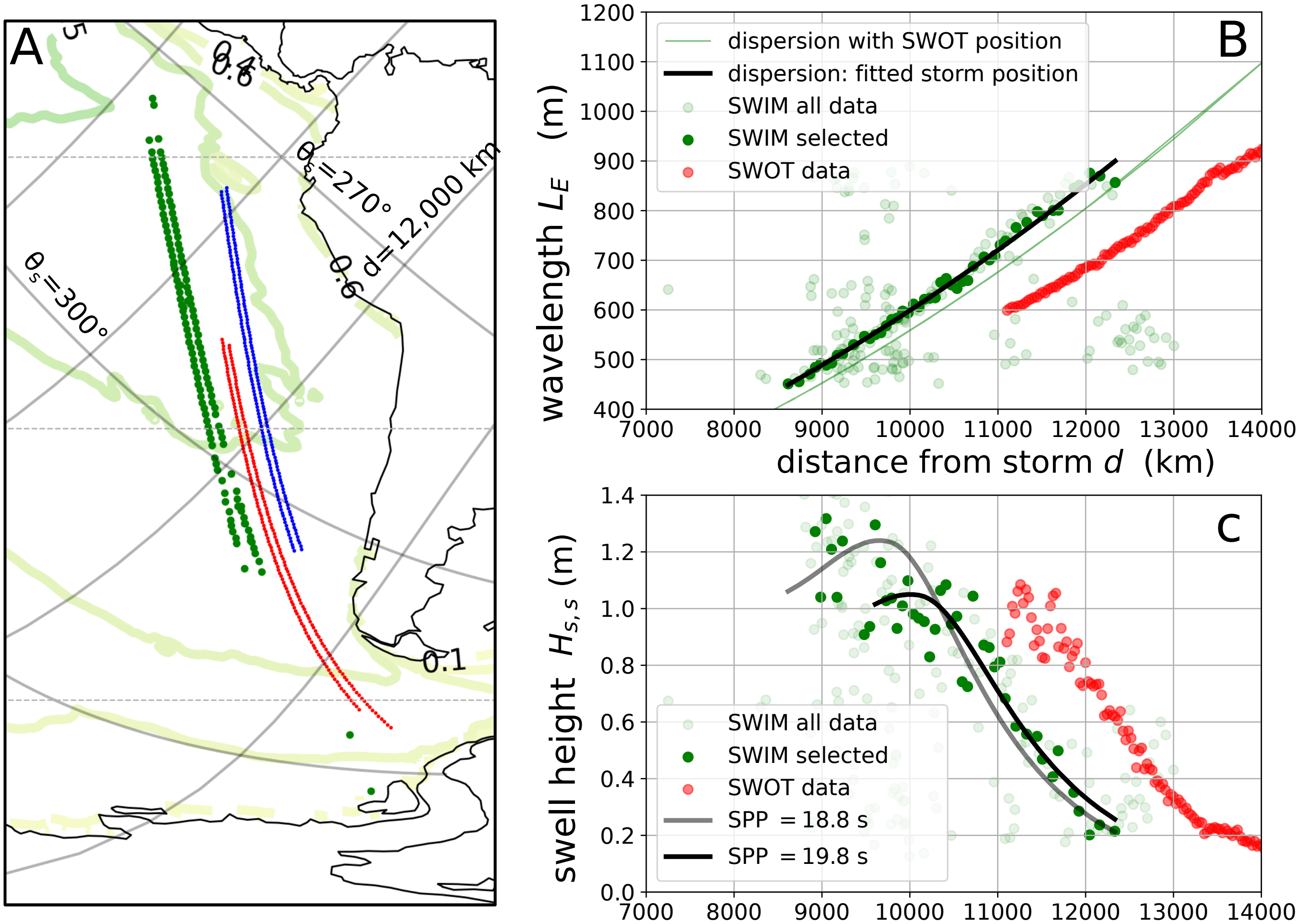

Fig. S2. (A) Map of Bolaven swell measurements by the 10° incidence beam of CFOSAT/SWIM 10° (green dots) at 20231015T06 UTC, relative to the closest SWOT measurements (9 hours later in red, 15 hours earlier in blue). (B) Mean wavelengths in SWIM and SWOT partitions (C) swell heights from SWIM (green dots) and SWOT (red dots) and fitted swell height (grey and black curves) using either all good data or only data with $L_E > 550$ m. Note that the SWIM "all data" contains all partitions, including partitions from different storms, not just Bolaven. The data selection keeps only swell partitions with a direction within 15° from the expected swell arrival direction (based on the storm position).

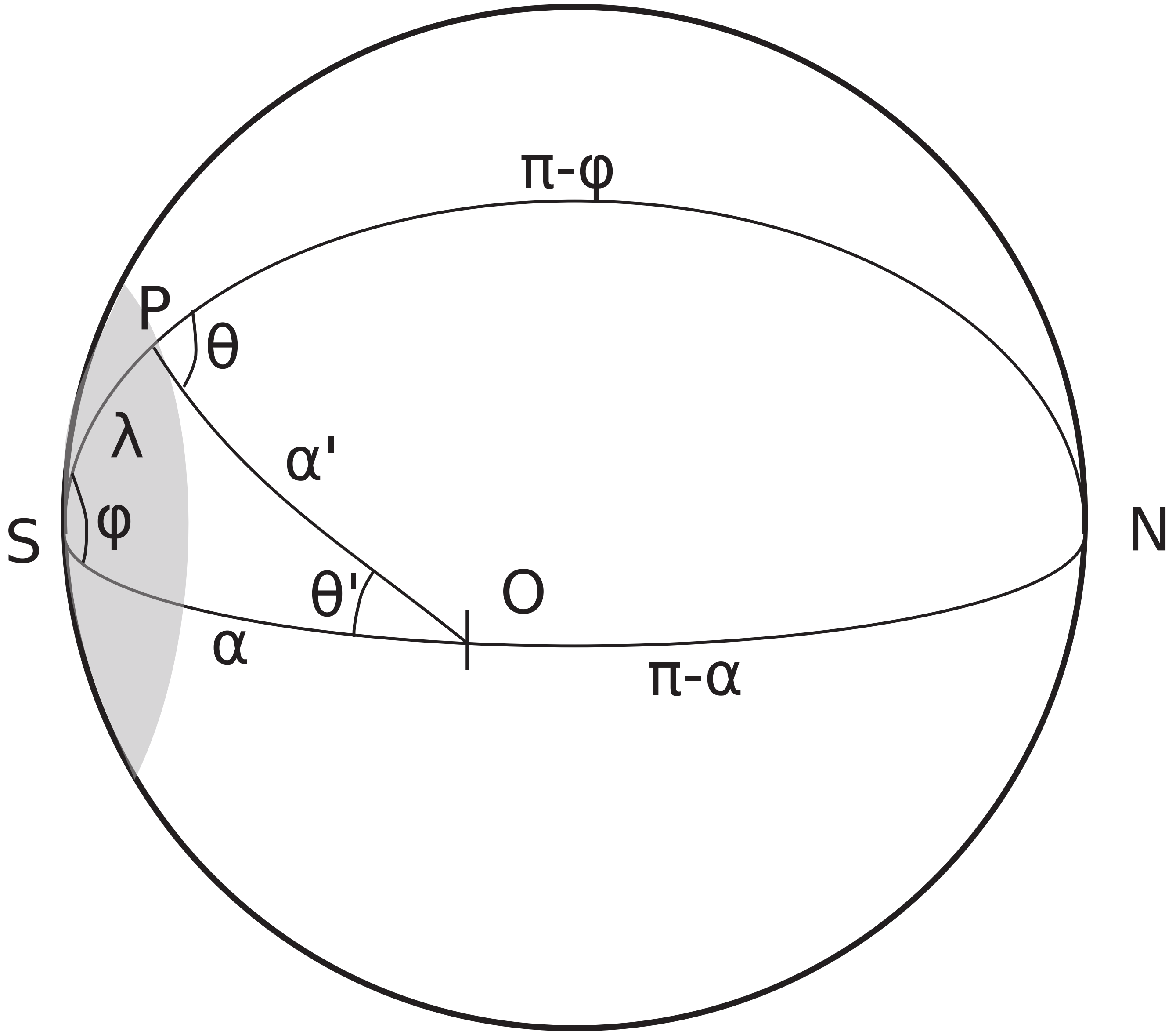

Fig. S3. Schematic of wave propagation on the sphere. The analytic propagation model is based on a uniform and isotropic wave spectrum within the shaded area (taken as a circular storm around the South pole S for simplicity). Waves present at point P at time t_S have propagated to the observation point O at time t_O.

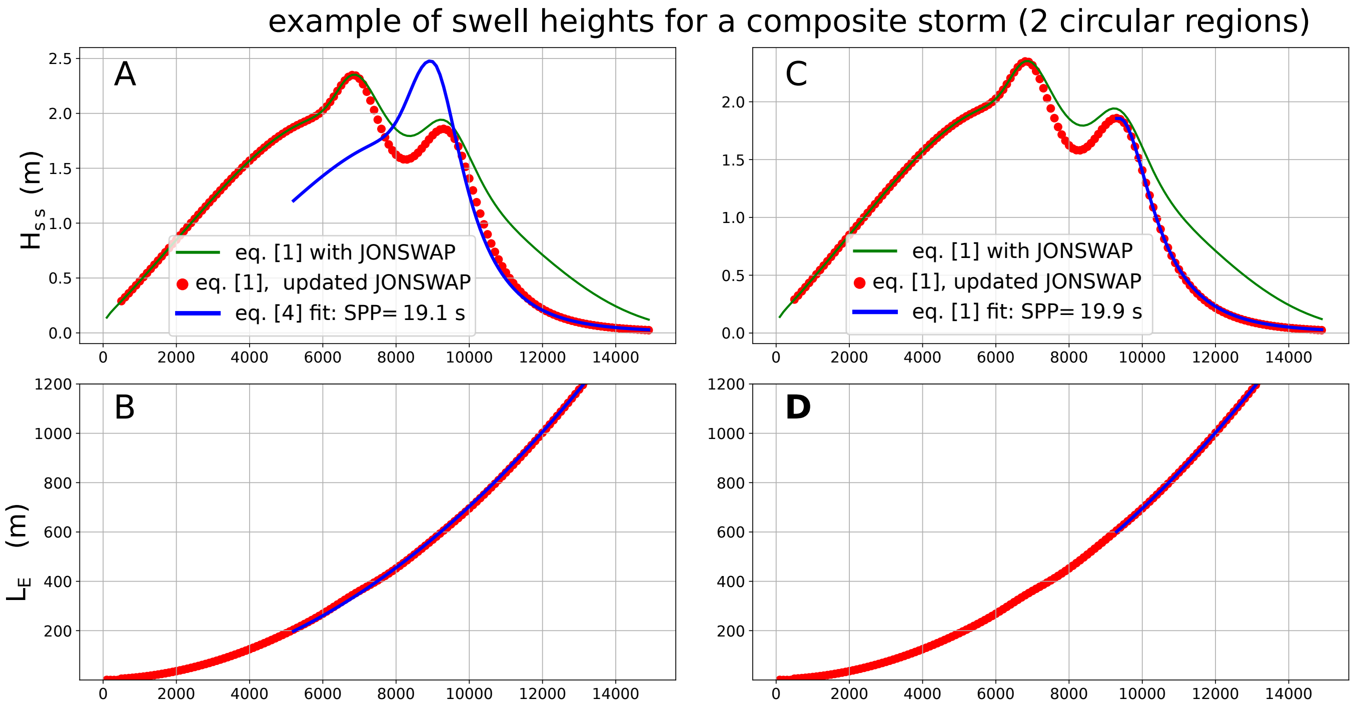

Fig. S4. Example of simulated swell height and wavelength using the analytic propagation model, for a composite storm that includes two regions. Region 1 with 300 km radius, centered at x=0, $T_p = 20$s, region 2 with 600 km radius, centered at $x = -300$ km, $T_p = 15.4$s. Note that the 2nd region covers the 1st region: when we combine the two, we remove energy from the second region in the intersection. The simulated data is fitted in the same way as the SWOT data in Fig. 2.E,F, using either, A,B a wide range of wavelengths or, C,D, a restricted range.

Movie S1. Visualization of modeled wave heights H_{18} (i.e. corresponding to the range of wavelengths L > 500 m typically resolved in SWOT Low Resolution data) and SWOT satellite tracks. The threshold at 5 cm is expected to be close to the threshold for swell detection by SWOT in most conditions. The model used here is described in ref. 15. Storm tracks for the top 400 storms, and Hs max values are overlaid over the map of H_{18}. The black crosses along the tracks indicate the position of the maximum along the track (one example is Fig. 2A). The animation illustrates how the generation of long period waves, in the model, occurs very close in time to the maximum H_s.

Movie S2. Visualization of modeled wave heights H_{25} (i.e. corresponding to the range of wavelengths $L > 975$ m typically resolved in SWOT Low Resolution data) and SWOT satellite tracks. The threshold at 5 cm is expected to be close to the threshold for swell detection by SWOT in most conditions. The model used here is described in ref. 15. Storm tracks for the top 400 storms, and Hs max values are overlaid over the map of H_{25}. The black crosses along the tracks indicate the position of the maximum along the track (one example is Fig. 2A). The animation illustrates how the generation of long period waves, in the model, occurs very close in time to the maximum H_s.

SI Dataset S1 (Dataset_S1.xslx)

This dataset provides the background data for SPP estimates from SWOT shown in Fig. 2c Storms are uniquely identified by their ranking in the Ardhuin-De Carlo storm catalogue, or for simplicity, by a name (e.g. ADC-0005 is called "Eddie"). SWOT data are defined by their cycle number, track number, and the side sub-swath which can be either left (L) or right (R). Before July 2023, for cycles above 300, SWOT is on a 1-day repeat orbit and the track positions are the same from one day to the next (see movie S1). After July 2023, the SWOT orbit repeats over each cycle that has a 21 day duration. From one day to the next, the track n+28 is shifted west by about 3° in longitude compared to track n of the previous day (e.g. red and blue tracks on Fig. 1.B). See movie S1 for all track positions. The selection criteria that define $L_{E,\min}$ and $L_{E,\max}$ are described in the Materials and Methods section. Values reported in the dataset S1 are a selection based on:

- SPP< 25 s,
- $\mathrm{mean}(H_{ss}) < 5$ m ,
- $L_{E,\max} > 700$ m,
- a Mean Absolute Percentage Error (MAPE) for the fitted H_{ss} less than 0.3,
- a MAPE for the fitted L_E less than 0.3,
- and a number of fitted values larger than 20.

After this first selection, for any given storm we discarded the tracks with the largest MAPE for H_{ss} and we excluded tracks for which the best fit distribution of L_E is shifted by more than one day from the a priori storm peak. The discarded tracks are highlighted in red, and the number that caused this is in bold. For each storm, a median and standard deviation of the Storm Peak Period is given in the header.

References

1. Hauser D, et al. (2021) New observations from the SWIM radar on-board CFOSAT: Instrument validation and ocean wave measurement assessment. *IEEE Trans. on Geosci. and Remote Sensing* 59(1):5–26.
2. Ifremer / CERSAT (2022) Global Ocean Directional Wave Parameters Level 2S from SWIM onboard CFOSAT for IWWOC project, version 1.0.
3. Hasselmann K, et al. (1973) Measurements of wind-wave growth and swell decay during the Joint North Sea Wave Project. *Deut. Hydrogr. Z.* 8(12):1–95. Suppl. A.
4. Krogstad HE, Wolf J, Thompson SP, Wyatt LR (1999) Methods for intercomparison of wave measurements. *Coastal Eng.* 37:235–257.
5. De Carlo M, Ardhuin F (2024) Along-track resolution and uncertainty of altimeter-derived wave height and sea level: re-defining the significant wave height in extreme storms. *J. Geophys. Res.* 134.
